# Chaotic and Stochastic Components in an Influenza Surveillance Series: Nonlinear Dynamics and Predictive Modeling Study

**DOI:** 10.2196/81547

**Published:** 2026-06-05

**Authors:** Carlos Pedro dos Santos Goncalves, Carlos Rouco

**Affiliations:** 1 ECEO - School of Organisational and Economic Sciences - Civil Aviation and Airport Management Department, Lusófona University, Intrepid Lab Hub of Lusófona University at CETRAD, Campo Grande, 376, Lisbon, 1749-024, Portugal, 351 217 515 500

**Keywords:** epidemiology, epidemiologic methods, epidemiological monitoring, chaos theory, topological data analysis, autoregressive conditional heteroskedasticity

## Abstract

**Background:**

Chaotic dynamics has been the subject of both theoretical and empirical research in epidemiology, with the most recent research strongly focusing on SARS-CoV-2. However, few empirical studies have been undertaken with respect to influenza, even though evidence of chaos has also been found in influenza surveillance data. Furthermore, empirical studies on chaos are focused on reconstructing hidden attractors in epidemiological time series to filter out noise; however, dynamical noise affecting chaotic dynamics can have relevant epidemiological features that are, in this way, left unresearched and that can be used for epidemiological surveillance and risk analysis by capturing the main underlying nonlinear processes associated with epidemiological dynamics.

**Objective:**

This study aimed to reinforce empirical research on chaotic dynamics in influenza surveillance and the study of the dynamical noise affecting that chaotic dynamics, addressing the consequences for epidemiological risk analysis and surveillance.

**Methods:**

Working with the weekly share of positive influenza tests for the Northern Hemisphere from January 2009 to March 2025 compiled by Our World in Data using FluNet data from the World Health Organization, we applied a recent method based on topological data analysis for reconstructing underlying attractors from time series and decomposing the dynamics down to independent and identically distributed noise. We adapted the method to the epidemiological context so that it can be used for predictive decomposition with direct application to epidemiological risk analysis and surveillance.

**Results:**

We found evidence of a low-dimensional chaotic attractor in the researched surveillance data, with a fractal dimension between 1 and 2, and a positive statistically significant largest Lyapunov exponent. The chaotic dynamics had power law scaling associated with long-wave influenza outbreaks, and it is affected by a stochastic component that is nonstationary in variance, leading to turbulent bursts in the outbreak dynamics. Testing different machine learning algorithms using the attractor as input for prediction and a 10-week rolling window, we found the following largest *R*^2^ scores for the prediction of the target series: 92.11% (1 week ahead), 85.95% (2 weeks ahead), 81.75% (3 weeks ahead), 77.59% (4 weeks ahead), and 73.35% (5 weeks ahead).

**Conclusions:**

The main results reinforce previous theoretical and empirical studies on chaos in epidemiology. Our findings showed that there is a 2-dimensional chaotic attractor that can support up to a 1-month prediction of the target surveillance series with high prediction scores and that the attractor plus noise can be modeled in a way that supports uncertainty quantification and epidemiological risk analysis.

## Introduction

Chaos theory deals with complex random-like dynamics in deterministic systems with strong sensitivity to small fluctuations that lead to exponentially divergent trajectories. In this way, apparent randomness can actually come from a system’s nonlinear dynamics rather than from any external noise source. From its early onset, chaos theory has been applied to epidemiological modeling with implications for the understanding of epidemiological dynamics linked to outbreak patterns and nonlinear viral contagion [1-8].

Among the first applications of chaos theory to epidemiology were studies of mathematical models when seasonality is introduced. An example of one such early application was the finding of chaotic dynamics in a susceptible-exposed-infective-recovered (SEIR) model [1]. In another early example [2], a nonlinear map was applied to an epidemiological modeling of parasite-host interactions leading to chaotic dynamics.

Although these examples from the 1980s already showed the possibility of chaos in theoretical mathematical epidemiological models, more recently, multiple theoretical studies have significantly expanded the scope and implications of chaos for epidemiology, with multiple examples of chaotic dynamics being found in different theoretical epidemiological models [3-8].

In [7], in particular, chaos was found in a susceptible-infective-recovered (SIR) model that incorporated government intervention and constrained health care capacity in the dynamical modeling, linking how constraints linked to health care capacity, nonlinear transmission, and public health care policy measures lead to complex dynamics including bifurcations and chaos with consequences for epidemiological outbreaks.

Although chaos has been found in theoretical epidemiological models, suggesting the possibility of chaos playing a relevant role in epidemiological modeling and understanding epidemiological dynamics, with possible consequences for health care policy, empirical studies on chaos have also been conducted. These have tried to find chaos in epidemiological data, with a particular emphasis during the COVID-19 period, when an abundance of epidemiological data allowed for the application of both theoretical and empirical methods from chaos theory [9-15].

The main approach of empirical studies into chaos is to reconstruct dynamical chaotic attractors from time series data and test for the presence of chaos. The objective, in the epidemiological context, is to uncover possible emergent low-dimensional chaotic attractors resulting from complex nonlinear epidemiological dynamics that can be used to both study and understand epidemiological patterns as well as for prediction, surveillance, and risk analysis.

In terms of empirical studies, a major finding in [13], with epidemiological relevance for health care, was the coexistence of chaos and quasiperiodic structures in epidemiological surveillance data from COVID-19.

Namely, in [13], methods for reconstructing chaotic attractors from time series were applied to mortality data from Turkey, Germany, Italy, and the United Kingdom, uncovering the presence of low-dimensional chaos in all analyzed countries with markers of quasiperiodicity in subseries. This is consistent with an emergent deterministic dynamics close to the onset of chaos, that is, close to a bifurcation point between periodic dynamics and chaotic dynamics.

This means that, although some deterministic process with chaotic dynamics is present and can be linked to outbreak waves, there are strong recurrences in surveillance data that can be exploited both for extracting information on the underlying chaotic dynamics and for prediction.

In [11], similar low-dimensional chaotic attractors were found for both deaths and number of positive COVID-19 cases in different world regions with the exception of Oceania, where a bifurcation from a less turbulent to a more turbulent chaotic regime was found and linked to the surfacing of the Delta and Omicron variants along with the ease in lockdown restrictions.

In [11], the chaotic dynamics was a form of chaos called color chaos that has a power law decay in the power spectrum and is linked to chaotic intermittency [11,16,17]. Similar patterns, with direct implication for health care management and policy, were found in daily hospital occupancy from COVID-19 in the United States and Canada [12], with high long-range predictability of hospital occupancy using the reconstructed chaotic attractors. This reinforced the findings in [11,13] but, in this case, for a time series linked to hospitalization.

Similar results were found for China [14], but, in this case, they were linked to an epidemiological rogue wave pattern that was found to be associated with a 3-dimensional chaotic attractor with a divergent branch structure. This attractor accounted for the rogue wave outbreak dynamics, and an explanation for these dynamics was linked to accelerating waves of viral contagion in urban environments of high population density.

These results have also been reinforced by epidemiological modeling, as addressed in [18], in which recurrence associated with seasonal waves alongside human population mitigation measures can lead to a delayed behavioral feedback loop that can trigger infection waves. The study also found that the interplay between seasonal epidemiological dynamics and human adaptive measures can lead to multiple attractors and chaos. The authors also found that the empirical data on COVID-19 showed evidence of this type of dynamics.

The bulk of studies and abundance of data on COVID-19 favored applications of empirical methods from chaos theory to SARS-CoV-2 surveillance data, and multiple authors uncovered positive evidence of chaos. There are fewer studies on seasonal influenza.

Indeed, although there have been empirical studies finding evidence of chaos in influenza surveillance data [19], the main focus has been on theoretical models, with the most recent studies applying empirical methods of chaos theory mainly focusing on SARS-CoV-2. This is due to the high availability of large publicly available sets of empirical data and the high level of surveillance that was implemented regarding the pandemic. Our aim in this work was to reinforce empirical research with possible chaotic dynamics in influenza.

Another major gap in the empirical study of chaos is that it usually focuses on the identification of an attractor in time series data affected by dynamical noise, in which the filtered noise is usually left out of the analysis focusing on the chaotic dynamics.

However, in chaotic dynamics, the presence of a stochastic component matters. Noise can both induce a transition from periodic dynamics to chaos (noise-induced chaos) and lead to periodic signatures (order induced by noise); thus, the theoretical study of stochastic chaos leads one to consider the need to account for both the chaotic dynamics linked to the nonlinear deterministic component and the stochastic dynamics itself [20-25].

In addition, in complex systems, there is the possibility of emergent chaotic dynamics being affected not by white noise but by more complex stochastic processes. In this way, recent empirical applications aimed at studying stochastic chaos have used topological data analysis to both find the set of parameters needed to reconstruct phase space attractors in noisy data and to decompose the resulting dynamics into both the chaotic and the stochastic dynamics. Studying both dynamics has direct implications for risk management [24,25].

In this work, we used such an approach for an influenza surveillance time series to study both the patterns associated with the possible chaotic attractor and the nature of any underlying stochastic process affecting that chaotic attractor. We also discuss their epidemiological implications for prediction, surveillance, and risk analysis.

In order to study both chaotic and stochastic dynamics in the series, we used a recent decomposition method that aims to study joint chaotic and stochastic dynamics using topological data analysis and machine learning [24,25]. The method was originally presented at the 4th International Conference on AI Research [25], with the results of an application for airport traffic leading to uncovering a low-dimensional chaotic attractor and a multifractal stochastic process that underwent a structural change after the COVID-19 disruption to air traffic.

This method was also applied to the study of sunspot dynamics, which led to the uncovering of two chaotic attractors operating on different scales plus a stochastic autoregressive process effectively decomposing the resulting dynamics down to independent and identically distributed (IID) noise [24].

Although this method aims to uncover complex attractors and decompose the dynamics down to IID noise for researching both possible chaotic and stochastic nonlinear dynamics, we adapted the method to incorporate a predictive decomposition modification to better fit epidemiological surveillance.

## Methods

### Data

The surveillance data we worked with were the weekly shares of positive influenza tests for all influenza types in the Northern Hemisphere from January 5, 2009, to March 31, 2025.

The data source is from Our World in Data, which processed the data from FluNet by the World Health Organization (2023), and the data are available under a Creative Commons license at the Our World in Data website [26].

The data are an aggregate indicator on the share of positive tests for the Northern Hemisphere calculated by the Our World in Data team from the FluNet original source and are part of the influenza data tracker made available at the Our World in Data website for researchers.

Even though the actual number of potential cases may individually be underestimated and country-specific data vary in accordance with the testing frequencies and protocols, the aggregate number calculated by the Our World in Data for the Northern Hemisphere as a whole mitigates, to some extent, the different testing frequencies and protocols by different countries. This makes it a surveillance series that is correlated with actual influenza outbreak processes and can provide a useful first-start, exploratory surveillance data series for studying the possibility of complex chaotic and stochastic epidemiological dynamics in influenza. This allows the application of the predictive decomposition method with epidemiological interpretability, as we show in both the Results and Discussion sections.

### Method Overview

**Figure 1.**
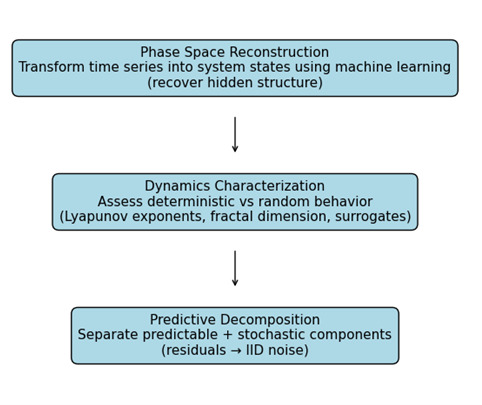
3-stage workflow plus outcomes. IID: independent and identically distributed.

The analysis followed a 3-stage workflow (Figure 1), which we describe in the following paragraphs.

### Step 1: Phase Space Reconstruction

The predictive phase space reconstruction method using topological machine learning is a critical step and is explained in detail in Multimedia Appendix 1. In this method, the observed time series was transformed into a geometric representation of system states using delay embedding in a way that maximizes prediction [27].

#### Objective

This step was necessary because the original 1-dimensional series did not directly capture the underlying dynamics. By reconstructing the trajectory in a higher-dimensional space, it became possible to identify relationships between past and future states that support prediction.

#### Hypothesis

The hypothesis was that there is an underlying attractor that can be used to predict the target series.

#### Statistical Techniques

We performed a preliminary analysis of the time series including the power spectrum necessary for characterization of the dynamics and used rolling window *k*-nearest neighbors as a search algorithm to find the optimal embedding parameters with the highest prediction performance. Plots of the recorded performance for different embedding parameters to evaluate the robustness of the possible attractor were also used.

#### Output

A reconstructed trajectory in a multidimensional geometric space captures the underlying dynamics and can be used for studying and predicting the epidemiological series.

### Step 2: Dynamics Characterization

The reconstructed trajectory was analyzed to determine whether it exhibited evidence of chaos or random behavior. This step established whether the observed dynamics contained meaningful structure or were primarily driven by noise.

#### Objective

This step was necessary to investigate the presence of random-looking dynamics that actually came from a chaotic attractor, since chaos allows for predictability within limits, using the attractor’s recurrence structure.

#### Hypothesis

The underlying series had some form of chaotic dynamics, and, if not, we had to characterize the type of dynamics in question.

#### Statistical Techniques

Tests for chaos were implemented to evaluate the hypothesis that we were dealing with a chaotic attractor and to characterize the type of chaotic attractor. This included fractal dimensions calculations, Lyapunov spectrum estimation, and bootstrap tests [28-34].

#### Output

The output was the characterization of possible complex nonlinear dynamics including its connection to underlying epidemiological factors and processes and implications for forecasting and surveillance.

### Step 3: Predictive Decomposition

The series was decomposed into a predictable component and a stochastic component. This step was necessary because epidemiological dynamics combine structured dynamics with random fluctuations. The decomposition was carried out until the residuals behaved as IID noise, meaning that no systematic linear nor nonlinear patterns remained, and the remaining variability was effectively random.

#### Objective

This step was necessary since chaotic attractors are often affected by complex stochastic processes, so identifying and characterizing the attractor were insufficient for prediction and risk analysis.

#### Hypothesis

The underlying series had some form of complex stochastic dynamics that can be captured and used for risk analysis.

#### Statistical Techniques

Different machine learning prediction algorithms were used, and the reconstructed attractor was used to predict the deterministic chaotic component. The residuals were then studied and modeled. The specific techniques depended on the type of residuals involved. In our case, we found the presence of nonstationary variance associated with time-varying nonlinear volatility, so methods adapted to deal with this type of process were used [35-40]. The Brock, Dechert, and Scheinkman (BDS) test was used to test for the presence of IID noise [41].

#### Output

A full decomposition down to IID noise was obtained, with the final result from all 3 stages of the method being a forecasting and epidemiological risk analysis system that can be used in epidemiological surveillance.

## Results

### Phase Space Reconstruction

**Figure 2.**
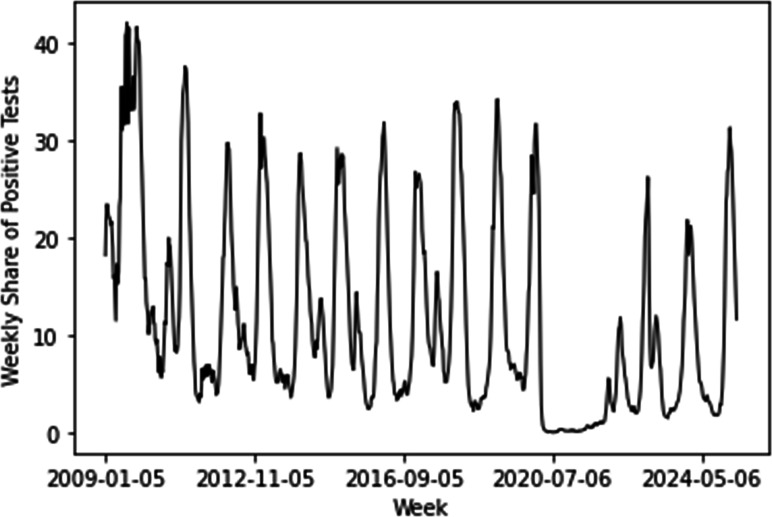
Weekly share of positive tests for influenza in the Northern Hemisphere, reported as percentage values. Source: FluNet by the World Health Organization (2023) and processed by Our World in Data from January 5, 2009, to March 31, 2025.

The weekly share of positive influenza tests showed an intermittent pattern, with long low-activity periods followed by sharp outbreak peaks (Figure 2). This indicates that influenza dynamics are not uniform over time but instead occur in bursts consistent with epidemic waves.

**Figure 3.**
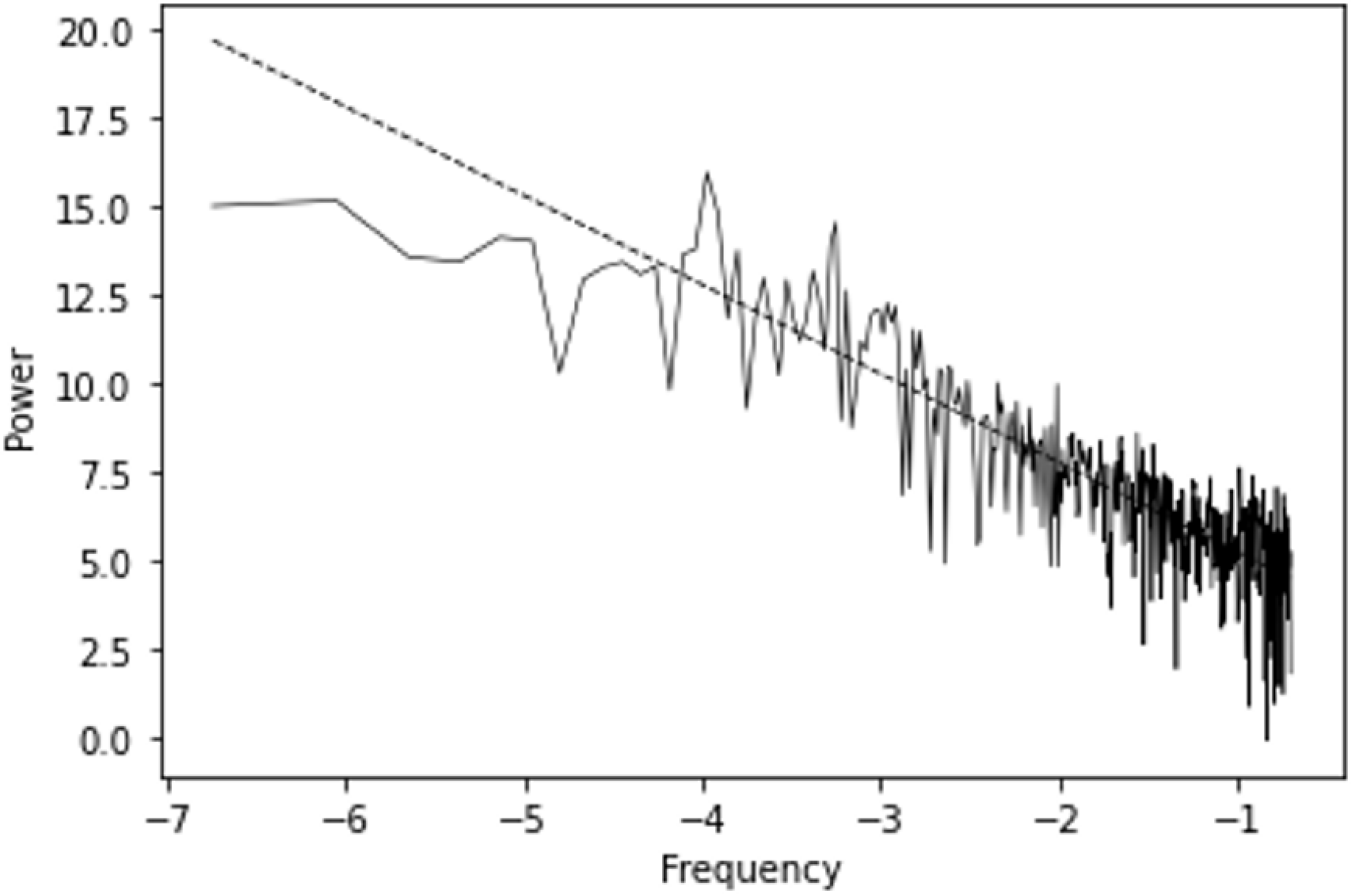
Power spectrum for Figure 2 series along with the fitted slope.

The power spectrum exhibited a power-law decay with a slope of −2.52 (*R*^2^=69.3%, *P*<.001; Figure 3). This showed that the series has strong persistence, meaning past values influence future behavior over extended periods [11,28]. This last result excluded the hypothesis of white chaos and indicated that, if chaos is present, it will be a form of color chaos [16].

Using a *k*-nearest neighbors’ regressor with a 10-week rolling window, the optimal embedding was obtained for dimension 2 and lag 11, achieving *R*^2^=78.33%, explained variance of 78.34%, correlation with the target series of 0.8902, and a root mean square error (RMSE) of 4.7277, which represented 11.23% of the series’ amplitude (Table 1). This indicated that a low-dimensional representation is sufficient to capture most of the system’s dynamics in a way that can support epidemiological prediction.

**Table 1.** Optimal embedding parameters and performance metrics obtained for a *k*-nearest neighbors’ regressor with a 10-week rolling window, 5 nearest neighbors, and *k*-dimensional search tree.

Results	Values
Lag	11
Dimension	2
*R*^2^ score, %	78.33
Explained variance, %	78.34
Correlation	.8902
RMSE^a^	4.7277
RMSE/amplitude, %	11.23

a RMSE: root mean square error.

**Figure 4.**
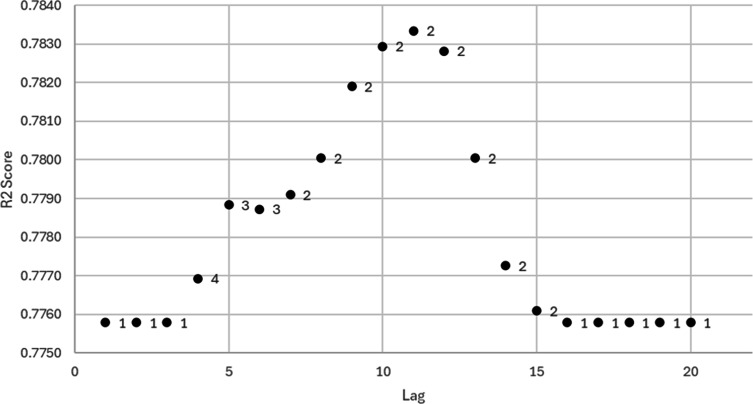
Highest *R*^2^ score as a function of the tested lag; the labels are the corresponding optimal dimensions for which the score was obtained.

**Figure 5.**
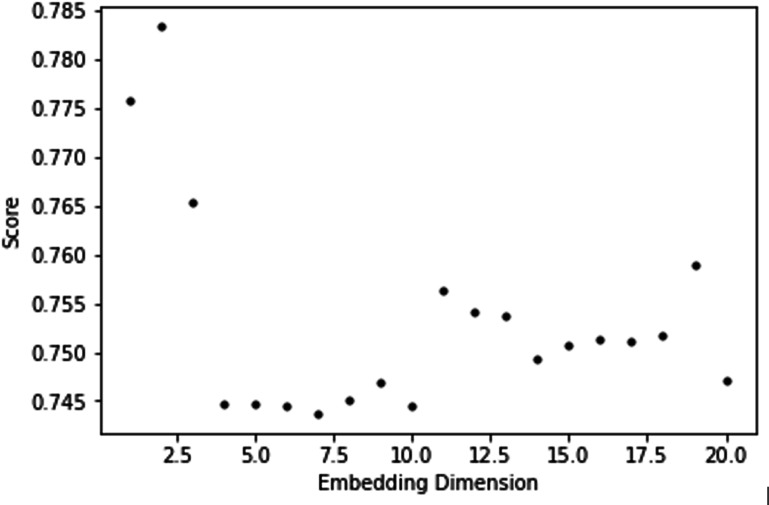
*R*^2^ score as a function of the embedding dimension for the lag 11.

To test the robustness of underlying 2-dimensional deterministic dynamics linked to influenza that can be used to predict the target series with a high prediction score, we needed to plot the optimal dimensions obtained for different lags (Figure 4) and, for the optimal lag, plot the score for different dimensions (Figure 5).

From Figure 4, we concluded that prediction performance exceeded 77% across all tested embeddings, which showed strong predictability using phase space embedding. We also found that dimension 2 was the most frequently selected, which demonstrated the robustness of the 2-dimensional phase space representation.

Performance decreased as embedding dimension increased beyond 2 to 3 (Figure 5); therefore, increasing the embedding dimension did not improve representation and may have reduced predictive accuracy. This indicated that the 2-dimensional structure was better at capturing the underlying epidemiological dynamics.

**Figure 6.**
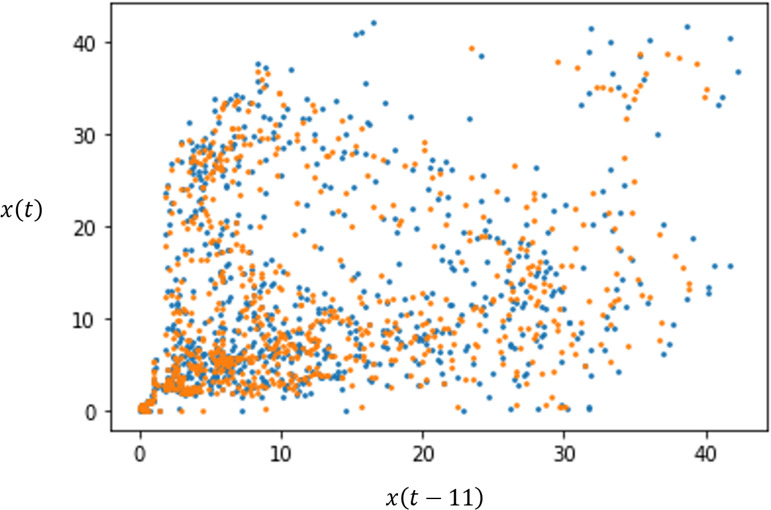
Phase space embedding of the original series (blue) and of the *k*-nearest neighbors’ predictions (orange).

Figure 6 shows the phase portrait of the trajectories. The reconstructed attractor exhibited a conic shape with a concentration of points near the origin. This showed that the system spends extended periods in low-activity states, with more dispersed trajectories corresponding with higher-activity phases and with the epidemiological outbreaks.

In Figure 6, there is an overall coincidence of the original reconstructed attractor and the *k*-nearest neighbors’ predicted trajectory, which further reinforced the aforementioned findings of strong predictability and that there was a strong deterministic feature to the dynamics.

### Dynamics Characterization

The largest Lyapunov exponent (LLE), estimated using the method by Eckmann et al [29], was positive for both the original and predicted attractors (7.1^–04^ and 9.1^–04^, respectively). The Kaplan-Yorke dimension lies between 1 and 2 (1.8587 for the original attractor and 1.9778 for the predicted attractor). The box-counting dimension estimates were in close agreement with the Kaplan-Yorke dimension (1.7891 and 1.8032, respectively; *P*<.001), which confirmed the presence of a fractal chaotic attractor.

Both estimated LLEs were statistically significant at the 1% level based on surrogate testing using the reconstructed, predicted, and wavelet-filtered attractors for the power law surrogate, excluding a power law noise process. For the stochastic version of the Iterative Amplitude Adjusted Fourier Transform (SIAAFT), the original reconstructed attractor’s LLE was statistically significant at the 5% level, with both filtered attractor’s LLEs statistically significant at the 1% level (Table 2).

**Table 2.** Surrogate tests for the largest Lyapunov exponent with bootstrap *P* values.

Results	Original reconstructed attractor	*k*-nearest neighbor filtered attractor	Daubechies 4 with 5-level wavelet filtered attractor
Estimated LLE^a^	7.1086^–04^	9.1216^–04^	1.0066^–03^
*P* value (power law surrogate)	.007	.003	.001
*P* value (SIAAFT^b^)	.011	.006	.001
N surrogates	999	999	999

a LLE: largest Lyapunov exponent.

b SIAAFT: stochastic version of the Iterative Amplitude Adjusted Fourier the Iterative Amplitude Adjusted Fourier Transform.

These results strongly reinforced the evidence that we were dealing with a chaotic attractor, rather than a purely random process. This implied that the dynamics retained an underlying deterministic structure that can be used for short- to medium-term prediction.

### Predictive Decomposition

Using the rolling window *k*-nearest neighbors’ predictions as the benchmark, we then compared the performance of different major machine learning–based predictive algorithms on a 10-day rolling window [42,43].

Ridge regression (λ=0.5) achieved the highest performance for 1-week-ahead prediction (*R*^2^=92.11%, RMSE=2.85; Table 3). This showed that short-term dynamics can be accurately approximated using rolling window linear models (Table 3).

**Table 3.** Prediction performance for a rolling window of 10 days for the main tested regression algorithms.

Regression algorithms	*R*^2^ score, %	Explained variance, %	RMSE^a^
KNN^b^ (k=5)	78.33	78.34	4.7277
SVM^c^ (radial basis function)	53.32	53.34	6.9395
SVM (linear)	91.74	91.79	2.9190
SVM (sigmoid)	31.47	31.49	8.4083
SVM (poly-2)	88.84	89.50	3.3931
SVM (poly-3)	80.59	81.06	4.4753
Ridge regression (0)	91.99	92.02	2.8739
Ridge regression (0.5)	92.11^d^	92.14^d^	2.8535^d^
Ridge regression (1.0)	92.04	92.08	2.8647
Random forests (10)	82.96	82.96	4.1924
Random forests (100)	83.10	83.10	4.1760
GBR^e^ (10)	76.42	76.42	4.9324
GBR (100)	85.90	85.91	3.8137

a RMSE: root mean square error.

b KNN: *k*-nearest neighbor.

c SVM: support vector machine.

d Optimum value in the comparison of the machine learning models, that is the maximum values for the *R*2 score and explained variance and the minimum value for the RMSE.

e GBR: gradient boosting regressor.

For multistep prediction, gradient boosting performed best for longer horizons (Table 4). This indicated that more complex nonlinear patterns became relevant as the prediction horizon increased. Prediction accuracy remained greater than 73% at 5 weeks (Table 4). This showed that the system retained predictive structure beyond short-term horizons.

**Table 4.** *R*^2^ scores for the rolling window learners in a 1- to 5-week-ahead prediction.

Regression algorithms	1 week, %	2 weeks, %	3 weeks, %	4 weeks, %	5 weeks, %
KNN^a^ (k=5)	78.33	76.16	73.88	71.51	69.05
SVM^b^ (radial basis function)	53.32	50.75	48.51	46.10	44.65
SVM (linear)	91.74	84.63	76.74	69.22	64.49
SVM (sigmoid)	31.47	31.78	32.06	32.20	32.15
SVM (poly-2)	88.84	79.65	70.72	60.56	55.85
SVM (poly-3)	80.59	71.14	65.70	52.24	44.34
Ridge regression (0)	91.99	85.57	79.24	73.93	69.96
Ridge regression (0.5)	92.11^c^	85.95^c^	79.86	74.65	70.94
Ridge regression (1.0)	92.04	85.94	79.90	74.63	70.83
Random forests (10)	82.96	81.78	79.40	75.91	72.43
Random forests (100)	83.07	81.67	79.29	75.79	72.17
GBR^d^ (10)	76.42	76.09	73.70	70.78	67.56
GBR (100)	85.90	84.74	81.75^c^	77.59^c^	73.35^c^

a KNN: *k*-nearest neighbor.

b SVM: support vector machine.

c Maximum *R*2 score value in the comparison of the machine learning models for each week-ahead prediction.

d GBR: gradient boosting regressor.

**Figure 7.**
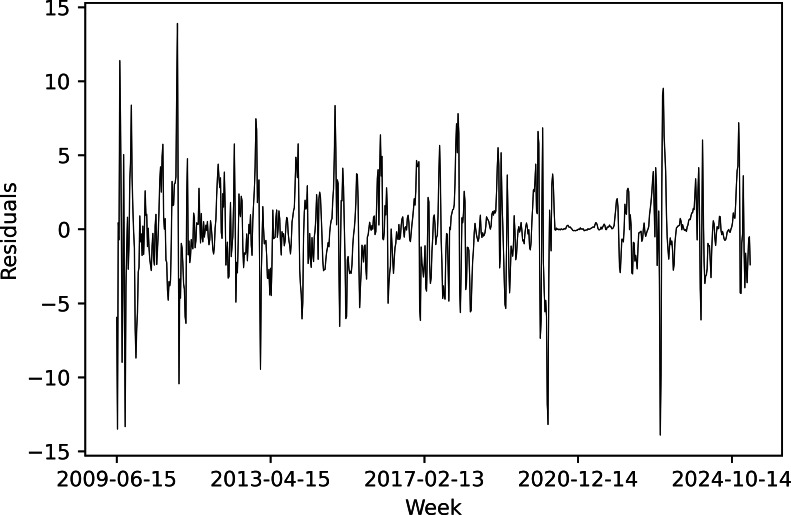
Residuals from the ridge regression predictor with 0.5 penalty.

Residuals from the ridge regression model showed clustering of large fluctuations in bursts (Figure 7), indicating time-varying volatility. The Engle test confirmed the presence of autoregressive conditional heteroskedasticity (ARCH) effects (Table 5), meaning that periods of high and low variability clustered over time rather than occurring randomly.

**Table 5.** Engle test for original residuals versus the noise term *z* from the exponentially weighted moving average (EWMA) model with *lambda* parameter ranging from .05 to .2 in steps of .05.

Parameters	Engle (LM^a^)	*P* value	Engle (*F*)	*P* value
Original residuals	200.8157	2.14^–29^	10.3762	1.81^–34^
Lambda=0.05	17.6846	.8555	.6998	.8606
Lambda=0.1	15.4182	.9311	.6083	.9345
Lambda=0.15	15.3453	.9329	.6054	.9363
Lambda=0.20	18.5512	.8181	.7349	.8238

a LM: Lagrange multiplier.

After applying an exponentially weighted moving average model (λ=0.1; Multimedia Appendix 2), no ARCH effects remained (Table 5). This indicated that volatility was largely captured by the model and occurred in short-lived bursts rather than persistent regimes [35-40].

The standardized residuals passed the BDS test for independence (Table 6) [41]. This showed that the remaining noise behaved as an approximately independent process.

**Table 6.** Brock, Dechert, and Scheinkman (BDS) test for original residuals versus the noise term *z* from the exponentially weighted moving average (EWMA) model with *lambda* parameter ranging from 0.05 to 0.2 in steps of 0.05 and embedding dimensions ranging from 2 to 10.

BDS test	Lambda=0.05	Lambda=0.1	Lambda=0.15	Lambda=0.20
d2	.5496	.2459	.0016	3.77E-08
d3	.1389	.9310	.0998	1.74E-04
d4	.0434	.4281	.4187	4.77E-03
d5	.0295	.2768	.6853	.0208
d6	.0205	.2006	.8629	.0462
d7	.0122	.1136	.8617	.1023
d8	.0174	.1286	.8616	.1223
d9	.0214	.1381	.8299	.1581
d10	.0473	.2216	.9507	.1459

The residual distribution was consistent with a Student *t* distribution (Table 7). This indicated the presence of heavy tails, meaning extreme deviations were more likely than under a normal distribution.

**Table 7.** Kolmogorov-Smirnov distribution test for the noise term *z*, for the Gaussian, Student *t,* and Cauchy distributions.

Results	Statistic	*P* value
Gaussian	.0834	2.24^–05^
Student *t*	.0374	.2004
Cauchy	.0769	1.21^–04^

None of the major generalized ARCH models estimated on the full sample passed the BDS test (Table S1 in Multimedia Appendix 2), which indicated that the exponentially weighted moving average model performed better at capturing the volatility pattern.

**Figure 8.**
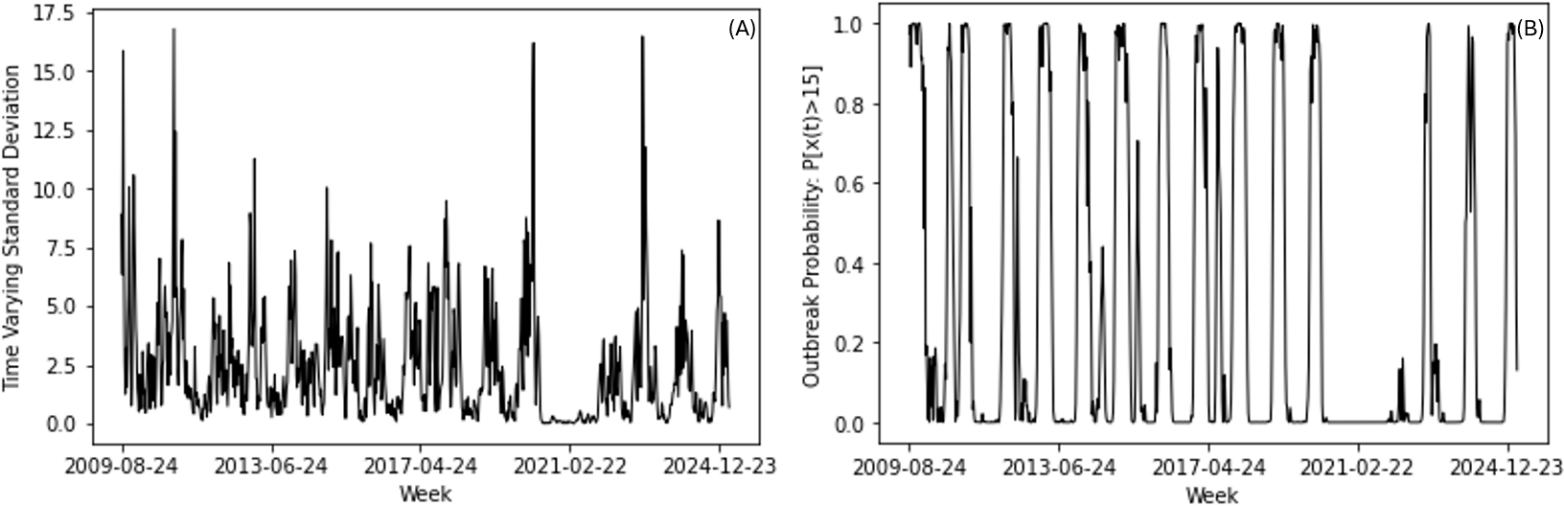
(A) Time-varying standard deviation for the target influenza surveillance series and (B) estimated outbreak probability using the predicted chaotic mean and the predicted variance for outbreak level of a share of positive tests higher than 15.

The estimated volatility exhibited burst-like increases over time (Figure 8A). This indicated that uncertainty rises during periods of higher influenza activity.

Outbreak probability varied over time as a function of both predicted levels and volatility as exemplified in Figure 8B for an outbreak event probability associated with a share of positive tests higher than 15. This showed that risk depended not only on expected activity but also on the level of uncertainty.

Periods of high influenza activity coincided with higher volatility. This suggests that outbreaks are associated with increased dispersion and elevated epidemiological risk.

## Discussion

### Principal Findings

In this work, we were able to decompose the dynamics of the weekly share of positive tests for influenza in the Northern Hemisphere from January 1, 2009, to March 31, 2025, into a component that is driven by a low-dimensional chaotic attractor characterized by black chaos with long-range persistence and a stochastic component that is the result of a time-varying volatility parameter multiplied by IID noise.

We now discuss the implications of the main results on 3 levels: the first level regards the connection to previous literature on theoretical epidemiological modeling and the implication in terms of reinforcing this literature and providing new directions for theoretical epidemiological modeling; the second level regards the connection to the previous empirical work on chaos in epidemiology including in influenza surveillance data; and the third level regards the implications for epidemiological risk analysis, monitoring, and surveillance.

### Implications for Theoretical Studies Into Chaos in Epidemiology

Considering the first level, our results reinforced the theoretical studies that have found chaotic dynamics to be possible in basic epidemiological models [1-8], while these models raised the hypothesis of chaos in general epidemiological dynamics, including the issue of whether chaos can actually be empirically present in these dynamics and what types of chaos are a key problem for empirical research on epidemiology.

A basic implication of chaos for epidemiology is that policymaking may have nontrivial, nonlinear consequences on the dynamics, including the possibility of triggering chaotic dynamics and turbulent bursts as addressed in [9], or even reduce them, as took place during the COVID-19 lockdown for the influenza series that we analyzed.

Another implication of chaos is that it has a complex relationship with predictability, in the sense that, although chaos implies limits to predictability due to the sensitive dependence upon initial conditions, chaotic attractors also have topological signatures that allow for predictability. In addition, there are different types of chaos, ranging from white chaos, which has a white noise–like spectrum and is less predictable, to color chaos, which has long-range dependence that makes it more predictable.

In our empirical results, the evidence was favorable to the hypothesis of color chaos in the form of black chaos, which is consistent with epidemiological models that exhibit chaotic intermittency associated with epidemiological seasonality and with previous studies using influenza data [19].

Although consistent with the theoretical possibility of chaos in epidemiological models, the decomposition down to IID noise surfaced an issue regarding these models. A major mathematical approach to the modeling of stochastic chaos usually involves adding some form of IID Gaussian or uniform noise to a deterministic set of equations that can exhibit deterministic chaos [20,22]. The findings from our decomposition method, however, did not support this assumption in the epidemiological context.

Indeed, we found that the stochastic dynamics associated with the noise factor in the predictive decomposition, rather than IID, showed a nonstationary variance with ARCH-like dependence. This means that epidemiological mathematical modeling that produces chaos in SIR and SEIR models needs to consider both the impact of simple IID noise. It also needs to compare it with what happens when stochastic processes with ARCH dependence in the variance are introduced, since these processes may lead to significant qualitative changes in the chaotic dynamics, namely, in the connection between outbreaks and turbulence.

Through the decomposition method, we found that outbreak waves visible in the surveillance series under study tended to occur in tandem with a rise in variance. This leads to a rise in dispersion in these periods as well as turbulence bursts associated with the outbreak waves.

The other impact of the ARCH stochastic component is that it injects short-run fast turbulence in the attractor’s long-range dynamics, leading to stochastic turbulence leaks into the chaotic dynamics.

The study of these phenomena in stochastic versions of general theoretical epidemiological models, like SIR and SEIR models, that exhibit deterministic chaos is a relevant research direction that may feed back into new empirical methods for epidemiological simulation, policymaking, and risk modeling.

### Empirical Implications

The second dimension of this work regards its connection to previous empirical works on chaos in epidemiological surveillance data. We found evidence of a low-dimensional fractal attractor with a positive LLE that was low but statistically significant upon surrogate testing on both the original and the noise-filtered series. The exponent after wavelet noise filtering was close to those found for COVID-19 surveillance data, though slightly smaller [11-14].

Regarding the influenza data, our work reinforced the findings in [19], in which statistically significant (upon surrogate testing) evidence of chaos was found in influenza data, with positive LLEs and long memory consistent with color chaos, which is also consistent with our findings.

However, by decomposing the attractor, we also found additional evidence regarding the dynamical noise affecting the influenza epidemiological dynamics that is nonstationary in variance. This result is new and has a significant implication, not only in the sense discussed in the previous paragraphs regarding the theoretical epidemiological studies but also for the type of ARCH dependence found.

Indeed, although, for instance, in financial time series, the ARCH stochastic markers are long range with strongly persistent volatility, in the influenza data, those markers showed a short-range dependence that was mainly driven by the autoregressive dependence upon the squared noise. This means that the nonstationary dynamics in the variance may be linked to short-range epidemiological factors associated with disease propagation in the population, which can introduce bursts in viral transmission during an outbreak process that amplifies the dispersion and the uncertainty during those outbreak waves.

The uncovering of the ARCH effect in the influenza surveillance is new and needs further research. Since empirical studies usually focus on uncovering the chaotic dynamics and filter out the noise not studying it, which is the empirical gap that the decomposition method that we applied addresses, further studies of decomposition for influenza and other viruses are relevant to determine whether ARCH dependence is a general feature.

Although the original decomposition method that we applied here was applied to air traffic and sunspot chaos [24,25] in order to model the stochastic component, in this work, the decomposition method was adapted to a direct predictive epidemiological surveillance scheme operating on the surveillance time series data. This was achieved by testing multiple prediction algorithms using a rolling window framework that is effective when dealing with small datasets and complex nonlinear dynamics such as what we were dealing with. This brings us to the third level of contribution of this work.

### Implications for Epidemiological Risk Analysis and Surveillance

There are two implications of the decomposition method for epidemiological risk analysis and surveillance. First, if we wish to extract outbreak probabilities, we need to account for both the chaotic component that affects the mean and the stochastic ARCH dynamics that affect the variance. In this way, epidemiological risk measures such as the standard deviation of the target epidemiological series and outbreak probabilities change with time, and this dynamics needs to be taken into account in epidemiological risk analysis.

The second implication for epidemiological risk analysis and surveillance regards the prediction of the time-varying mean of the process, which, in this case, results from the chaotic dynamics.

As we showed, a rolling window scheme with a machine learning unit can be used effectively to predict the mean and provide for an expectation of the target influenza surveillance series with a very high *R*^2^. However, there are a few caveats, as we now explain.

For the influenza surveillance target that we worked on, which is the weekly share of positive tests for influenza in the Northern Hemisphere from January 5, 2009, to March 31, 2025, we found that, for 1-week and 2-week-ahead predictions, using the reconstructed attractor as a predictor, the adaptive local linear ridge regressor achieved *R*^2^ scores of 92.11% and 85.95%, respectively. This means that the adaptive local linear approximation works well for 1 or 2 weeks; however, for 3 up to 5 weeks ahead, the ridge regressor is no longer the best performer.

In the compared models, we found the gradient boosting regressor with 100 estimators performed better than the rest for more than 2 weeks ahead, with *R*^2^ scores of 81.75%, 77.59%, and 73.35% for 3-week, 4-week, and 5-week-ahead predictions, respectively.

Another relevant point is that the topological structure of the attractor contains sufficient information in the *k*-nearest neighbor structure to allow for up to 5-week-ahead predictions with *R*^2^ scores of 78.33% (1 week), 76.16% (2 weeks), 73.88% (3 weeks), 71.51% (4 weeks), and 69.05% (5 weeks), which means that, overall, the topological structure of the reconstructed attractor supports epidemiological prediction for influenza. This has value for epidemiological planning that uses epidemiological surveillance variables such as the share of positive tests, with a high *R*^2^ for up to 1 month ahead.

### Limitations and Future Work

This study focused on a single surveillance series and region as a first exploratory step. Further studies are needed in multiple surveillance regions and different epidemiological series on influenza from a country level to a regional level. This will help achieve a deeper understanding and characterization of the influenza epidemiological dynamics regarding both the presence of chaotic dynamics and turbulent ARCH effect found in this series.

In particular, if both low-dimensional black chaos and similar ARCH dynamics are found in both hemispheres, transmission zones, and country-level data, then there is robust evidence for modeling influenza using stochastic chaos with ARCH dynamics incorporated in the noise variables.

Although black chaos is expected to be present due to the long-range intermittent process associated with viral outbreaks, the critical issue is the presence or absence of the ARCH dynamics in different surveillance series.

Similarly, for other viruses of interest, such as SARS-CoV-2, the application of the decomposition method is needed in order to uncover the type of deterministic and stochastic dynamics as well as the factors that may be present in different epidemiological data and epidemiological surveillance target variables. This is also needed to determine the implications for epidemiological surveillance, prediction, and risk analysis, since, although black chaos was also found for SARS-CoV-2, ARCH needs to be researched.

## Supplementary material

10.2196/81547 Multimedia Appendix 1 Phase space predictive topological reconstruction.

10.2196/81547 Multimedia Appendix 2 Autoregressive conditional heteroskedasticity model specification.

## Abbreviations

ARCH: autoregressive conditional heteroskedasticity
BDS: Brock, Dechert, and Scheinkman
IID: independent and identically distributed
KNN: *k*-nearest neighbors
LLE: largest Lyapunov exponent
RMSE: root mean square error
SEIR: susceptible-exposed-infective-recovered
SIAAFT: stochastic version of the Iterative Amplitude Adjusted Fourier Transform
SIR: susceptible-infective-recovered
SVM: support vector machine

## References

[1] Aron JL, Schwartz IB (1984). Seasonality and period-doubling bifurcations in an epidemic model. J Theor Biol.

[2] Rogers TD, Yang ZC, Yip LW (1986). Complete chaos in a simple epidemiological model. J Math Biology.

[3] Li J, Teng Z, Wang G, Zhang L, Hu C (2017). Stability and bifurcation analysis of an SIR epidemic model with logistic growth and saturated treatment. Chaos Solitons Fractals.

[4] Huang YJ, Huang HT, Juang J, Wu CH (2022). Multistability of a two-dimensional map arising in an influenza model. J Nonlinear Sci.

[5] Brugnago EL, Gabrick EC, Iarosz KC (2023). Multistability and chaos in SEIRS epidemic model with a periodic time-dependent transmission rate. Chaos.

[6] Gai C, Kolokolnikov T, Schrader J, Sharma V (2024). Recurrent and chaotic outbreaks in SIR model. Eur J Appl Math.

[7] Dutta S, Dutta P, Akhtar P, Samanta G (2025). Bifurcation analysis and chaotic dynamics in an SIR model with nonlinear incidence and constrained healthcare capacity. Chaos Solitons Fractals.

[8] de Carvalho J (2025). Double Hopf bifurcation and chaotic dynamics in a periodically-forced SIR model. Sci Rep.

[9] Mangiarotti S, Peyre M, Zhang Y, Huc M, Roger F, Kerr Y (2020). Chaos theory applied to the outbreak of COVID-19: an ancillary approach to decision making in pandemic context. Epidemiol Infect.

[10] Postavaru O, Anton SR, Toma A (2021). COVID-19 pandemic and chaos theory. Math Comput Simul.

[11] Gonçalves CP (2022). Low dimensional chaotic attractors in SARSCoV-2’s regional epidemiological data. Int J Swarm Evol Comput.

[12] Gonçalves CP (2023). Low dimensional chaotic attractors in daily hospital occupancy from COVID-19 in the USA and Canada. Int J Swarm Evol Comput.

[13] Yılmaz E, Aydıner E (2024). Chaotic and quasi-periodic regimes in the COVID-19 mortality data. Chaos Theory Applications.

[14] Gonçalves CP (2024). Epidemiological rogue waves and chaos-induced multifractal self-organized criticality in COVID-19. Int J Swarm Evol Comput.

[15] Calistri A, Francesco Roggero P, Palù G (2024). Chaos theory in the understanding of COVID-19 pandemic dynamics. Gene.

[16] Chen P (1996). A random walk or color chaos on the stock market? Time-frequency analysis of S&P indexes. Stud Nonlinear Dynamics Econometrics.

[17] Kaplan D, Glass L (1995). Understanding Nonlinear Dynamics.

[18] Wagner J, Bauer S, Contreras S, Fleddermann L, Parlitz U, Priesemann V (2025). Societal self-regulation induces complex infection dynamics and chaos. Phys Rev Res.

[19] Oluwole OSA (2017). Deterministic chaos, El Niño southern oscillation, and seasonal influenza epidemics. Front Environ Sci.

[20] Gonçalves CP (2025). Stochastic chaotic network vector fields. Int J Swarm Evol Comput.

[21] Frey M, Simiu E (1993). Noise-induced chaos and phase space flux. Physica D Nonlinear Phenomena.

[22] Kaneko K, Tsuda I (2001). Complex Systems: Chaos and Beyond.

[23] Cvitanović P, Artuso R, Mainieri R (2023). Chaos: Classical and Quantum.

[24] Gonçalves CP (2024). Topological machine learning and chaotic attractors decomposition – an application to sunspot chaos. Int J Swarm Evol Comput.

[25] Gonçalves CP, Rouco C (2024). Artificial intelligence, smart topological data analysis and chaos in business continuity management: the case of COVID-19 in Birmingham Airport. Proc 5th Int Conference AI Res ICAIR 2024.

[26] Weekly share of influenza tests that were positive. Our World in Data.

[27] Takens F, Rand D, Young LS (1981). Dynamical Systems and Turbulence Lecture Notes in Mathematics.

[28] Schroeder MR (1991). Fractals, Chaos, Power Laws: Minutes from an Infinite Paradise.

[29] Eckmann JP, Kamphorst SO, Ruelle D, Ciliberto S (1986). Liapunov exponents from time series. Phys Rev A.

[30] Davison AC, Hinkley DV (1997). Bootstrap Methods and Their Application.

[31] Theiler J, Eubank S, Longtin A, Galdrikian B, Doyne Farmer J (1992). Testing for nonlinearity in time series: the method of surrogate data. Physica D Nonlinear Phenomena.

[32] Good PI (2005). Permutation, Parametric and Bootstrap Tests of Hypotheses.

[33] Venema V, Ament F, Simmer C (2006). A stochastic Iterative Amplitude Adjusted Fourier Transform algorithm with improved accuracy. Nonlin Processes Geophys.

[34] Phipson B, Smyth GK (2010). Permutation P-values should never be zero: calculating exact P-values when permutations are randomly drawn. Stat Appl Genet Mol Biol.

[35] Engle RF (1982). Autoregressive conditional heteroscedasticity with estimates of the variance of United Kingdom inflation. Econometrica.

[36] Bollerslev T (1986). Generalized autoregressive conditional heteroskedasticity. J Econom.

[37] Nelson DB (1991). Conditional heteroskedasticity in asset returns: a new approach. Econometrica.

[38] Baillie RT, Bollerslev T, Mikkelsen HO (1996). Fractionally integrated generalized autoregressive conditional heteroskedasticity. J Econom.

[39] Francq C, Zakoian JM (2019). GARCH Models: Structure, Statistical Inference and Financial Applications.

[40] Morgan/Reuters JP (1996). RiskMetrics - Technical Document.

[41] Broock WA, Scheinkman JA, Dechert WD, LeBaron B (1996). A test for independence based on the correlation dimension. Econom Rev.

[42] Alpaydin E (2014). Introduction to Machine Learning.

[43] Géron A (2022). Hands On Machine Learning with Scikit Learn, Keras, and TensorFlow.

