# Supplementary material for: Chaotic and Stochastic Components in an Influenza Surveillance Series: Nonlinear Dynamics and Predictive Modeling Study
Source: JMIRx Med. 2026 Jun 5;7:e81547. doi: 10.2196/81547 (PMC13241054; doi:10.2196/81547)
Supplement: Multimedia Appendix 1 [file xmed-v7-e81547-s001.docx]

## Phase Space Predictive Topological Reconstruction

Given a time series $x_{t}$ that is a function of an unknown *m*-dimensional attractor $w_{t}=\left( w_{1}\left( t \right),w_{2}\left( t \right),..,w_{m}\left( t \right) \right)$, that is, $x_{t}=g\left( w_{t} \right)$, delay embedding involves reconstructing the attractor’s dynamics in *d*-dimensional Euclidean space by using an appropriate lag *h* and embedding dimension $d$ [1], leading to the reconstructed phase space trajectory $v_{t}=\left( x_{t-\left( d-1 \right)h},\ldots,x_{t-2h},x_{t-h},x_{t} \right)$.

There is a slight difference with respect to Takens approach as discussed in [2-5]. The algorithm is actually building an embedding that has the strongest topological structure linking the last phase point to the next series value in such a way a $d$-dimensional vector $v_{t}$ is found for approximating $w_{t}$ using the recurrence structure of the reconstructed trajectory and topological machine learning to find that surrogate. So that a predictive embedding scheme is being used.

Topological adaptive machine learning embedding involves selecting the embedding where the recurrence structure associated with *k*-nearest neighbors leads to the highest prediction performance in predicting the next value of the time series from the previous value of the phase point. In this way, a rolling window of size *w*, the training set is given by the tuples of the form:

| $X_{t-1}=\left\{ \left( v_{s-1},x_{s} \right):s=t-w-1,\ldots,t-2,t-1 \right\}$ | (A.1) |
| --- | --- |

In order to predict the next value of the series $x_{t}$, the phase point $v_{t-1}$, which is outside the training sample is supplied, the topological learner then finds the *k*-nearest neighbors’ of this phase point, using Euclidean distance, in the reconstructed trajectory within the training sample, the time indices for the *k* nearest neighbors are identified as:

| $N_{k,t-1}=\left\{ s_{1},s_{2},\ldots,s_{k} \right\},$ | (A.2) |
| --- | --- |

such that:

| $\left\Vert v_{t-1}-v_{s_{1}} \right\Vert_{2}\leq\left\Vert v_{t-1}-v_{s_{2}} \right\Vert_{2}\leq\ldots\leq\left\Vert v_{t-1}-v_{s_{k}} \right\Vert_{2}$ | (A.3) |
| --- | --- |

Which leads to the training data subset:

| $X\left( v_{t-1} \right)=\left\{ \left( v_{s_{i}},x_{s_{i}+1} \right) \right\}$ | (A.4) |
| --- | --- |

Then $x_{t}$ is predicted using the training data subset points through a Euclidean distance weighted average:

| $\hat{x}_{t}=\frac{\sum_{i=1}^{k} w_{i}x_{s_{i}+1}}{\sum_{i=1}^{k} w_{i}}$ | (A.5) |
| --- | --- |
| $w_{i}=\frac{1}{\left\Vert v_{t-1}-v_{s_{i}} \right\Vert_{2}}$ | (A.6) |

In this way, the prediction captures the local recurrence structure of the attractor. By searching for the embedding that leads to the highest prediction performance measured by the *R*^2^ score, from within a set of alternative embedding dimensions and lags we try to find the embedding where the recurrence structure in terms of *k*-nearest neighbors leads to the strongest exploitable topological information, this is the embedding where the attractor has the most clear topological structure linking the reconstructed attractor’s trajectory to the next value of the series.

In this way, given an unknown structural mapping $v_{t}=F\left( v_{t-1} \right)$, the machine learning algorithm is approximating the compound function using the $v_{t}$ as an approximation for the unknown $w_{t}$ operating as a surrogate vector for $w_{t}$:

| $x_{t}=g\left( v_{t} \right)=g\left( F\left( v_{t-1} \right) \right)=f\left( v_{t-1} \right)$ | (A.7) |
| --- | --- |

With the compound defined as $f=g\circ F$.

The algorithm in this way does not estimate a model, instead it uses the recurrence structure for prediction operating on the *k*-nearest neighbors for prediction, therefore, it provides for a topological baseline which is a critical point since in chaos there is an internal topological structure for prediction linked to the set of unstable periodic orbits that leaves a recurrence skeleton associated with the attractor’s topological structure, the *k*-nearest neighbors operates solely on topological information exploiting this skeleton; therefore, it provides for a baseline in which to find an underlying deterministic structure which is effective in chaotic dynamics affected by noise, it also provides for a forecasting baseline, one that uses solely the topological information, allowing one to assess the degree to which an attractor’s topological structure can be exploited [4-7].

**References**

1. Takens F. Detecting strange attractors in turbulence. In: Rand D, Young LS, editors. Dynamical Systems and Turbulence Lecture Notes in Mathematics. Vol 898. Springer-Verlag; 1981:366-381. [doi: 10.1007/BFb0091924] ISBN: 978-3-540-11171-9
2. Gonçalves CP. Low dimensional chaotic attractors in daily hospital occupancy from COVID-19 in the USA and Canada. Int J Swarm Evol Comput. 2023;11:291. [doi: 10.35248/2090-4908.22.11.291]
3. Gonçalves CP. Epidemiological rogue waves and chaos-induced multifractal self-organized criticality in COVID-19. Int J Swarm Evol Comput. 2024;13:367. [doi: 10.35248/2090-4908.24.13.367]
4. Gonçalves CP. Topological machine learning and chaotic attractors decomposition – an application to sunspot chaos. Int J Swarm Evol Comput. 2024;13:387. URL: <https://www.walshmedicalmedia.com/open-access/topological-machine-learning-and-chaotic-attractors-decompositionan-application-to-sunspot-chaos.pdf> [doi: 10.35248/2090-4908.24.13.387]
5. Gonçalves CP, Rouco C. Artificial intelligence, smart topological data analysis and chaos in business continuity management: the case of COVID-19 in Birmingham Airport. Proceedings of the 5th International Conference on AI Research, ICAIR 2024. 2024;5(1):507-516. [doi: 10.34190/icair.4.1.3329]
6. Kaplan D, Glass L. Understanding nonlinear dynamics. Springer-Verlag; 1995. URL: <http://link.springer.com/10.1007/978-1-4612-0823-5> [doi: 10.1007/978-1-4612-0823-5] ISBN: 0-387-94440-0
7. Cvitanović P, Artuso R, Mainieri R, et al. Chaos: Classical and Quantum. Niels Bohr Institute; 2023. URL: <https://chaosbook.org/> [Accessed 2026-05-28]
