# Supplementary material for: Chaotic and Stochastic Components in an Influenza Surveillance Series: Nonlinear Dynamics and Predictive Modeling Study
Source: JMIRx Med. 2026 Jun 5;7:e81547. doi: 10.2196/81547 (PMC13241054; doi:10.2196/81547)
Supplement: Multimedia Appendix 2 [file xmed-v7-e81547-s002.docx]

## Autoregressive Conditional Heteroskedasticity Models Specification

Below are the specifications for the main ARCH models employed, in the case of FIGARCH, Python’s ARCH library automatically estimates the parameter *d* for the fractional difference.

- EWMA: $\sigma_{t}^{2}=\lambda\sigma_{t-1}^{2}+\left( 1-\lambda\right)u_{t-1}^{2}$
- ARCH: $\sigma_{t}^{2}=c_{0}+c_{1}u_{t-1}^{2}$
- GARCH(1,1): $\sigma_{t}^{2}=c_{0}+c_{1}u_{t-1}^{2}+c_{2}\sigma_{t-1}^{2}$
- EGARCH(1,1): $\ln\sigma_{t}^{2}=c_{0}+c_{1}\left( \left| u_{t-1} \right|-\sqrt{\frac{2}{\pi}} \right)+c_{2}\ln\sigma_{t-1}^{2}$
- FIGARCH(1,1): $\sigma_{t}^{2}=c_{0}+\left( 1-c_{1}L-c_{2}L\left( 1-L \right)^{d} \right)u_{t}^{2}+c_{1}\sigma_{t-1}^{2}$

**Table S1:** Engle LM and F tests’ *P* values along with BDS test’s lowest *P* value applied to *z* obtained from the filtering of the dynamical volatility estimated for the major ARCH family models with the Student *t* distribution.

|  | Engle LM (*P* value) | Engle F (*P* value) | BDS (min *P* value) |
| --- | --- | --- | --- |
| GARCH(1,0) | 0.2001 | 0.1989 | 1.60E-53 |
| GARCH(1,1) | 0.0178 | 0.0163 | 3.01E-103 |
| EGARCH(1,1) | 0.0543 | 0.0520 | 1.79E-61 |
| GJR-GARCH | 0.0007 | 0.0005 | 2.76E-125 |
| FIGARCH(1,1) | 0.0178 | 0.0163 | 2.98E-103 |
